# Nimodipine systemic exposure and outcomes following aneurysmal subarachnoid hemorrhage: a pilot prospective observational study (ASH-1 study)

**DOI:** 10.3389/fneur.2023.1233267

**Published:** 2024-01-05

**Authors:** Sherif Hanafy Mahmoud, Fatma Hefny, Fadumo Ahmed Isse, Shahmeer Farooq, Spencer Ling, Cian O'Kelly, Demetrios James Kutsogiannis

**Affiliations:** ^1^Faculty of Pharmacy and Pharmaceutical Sciences, University of Alberta, Edmonton, AB, Canada; ^2^Pharmacy Services, University of Alberta Hospital, Alberta Health Services, Edmonton, AB, Canada; ^3^Vascular, Endovascular and General Neurosurgery, Division of Neurosurgery, Faculty of Medicine and Dentistry, University of Alberta, Edmonton, AB, Canada; ^4^Department of Critical Care Medicine, Faculty of Medicine and Dentistry, University of Alberta, Edmonton, AB, Canada

**Keywords:** nimodipine, aneurysmal subarachnoid hemorrhage, pharmacokinetics, vasospasm, delayed cerebral ischemia, modified Rankin scale

## Abstract

**Background:**

Nimodipine improves outcomes following aneurysmal subarachnoid hemorrhage (aSAH). Guidelines recommend that all patients should receive a fixed-dose nimodipine for 21 days. However, studies reported variability of nimodipine concentrations in aSAH. It is not clear if reduced systemic exposure contributes to worsening outcomes. The aim of this study was to compare nimodipine systemic exposure in those who experienced poor outcomes to those who experienced favorable outcomes.

**Methods:**

This was a pilot prospective observational study in 30 adult patients admitted to the University of Alberta Hospital with aSAH. Data were collected from the electronic health records following enrollment. Blood samples were collected around one nimodipine 60 mg dose at a steady state, and nimodipine [total, (+)-R and (−)-S enantiomers] plasma concentrations were determined. The poor outcome was defined as a modified Rankin Scale (mRS) score at 90 days of 3-6, while the favorable outcome was an mRS score of 0-2. The correlation between nimodipine concentrations and percent changes in mean arterial pressure (MAP) before and after nimodipine administration was also determined. Furthermore, covariates potentially associated with nimodipine exposure were explored.

**Results:**

In total, 20 (69%) participants had favorable outcomes and 9 (31%) had poor outcomes. Following the exclusion of those with delayed presentation (>96 h from aSAH onset), among those presented with the World Federation of Neurological Surgeons (WFNS) grade 3–5, nimodipine median (interquartile range) area under the concentration time curve (AUC_0-3h_) in those with favorable outcomes were 4-fold higher than in those with poor outcomes [136 (52–192) vs. 33 (23–39) ng.h/mL, respectively, value of *p* = 0.2]. On the other hand, among those presented with WFNS grade 1–2, nimodipine AUC_0-3h_ in those with favorable outcomes were significantly lower than in those with poor outcomes [30 (28–36) vs. 172 (117–308) ng.h/mL, respectively, value of *p* = 0.03)]. (+)-R-nimodipine AUC_0-3h_ in those who did not develop vasospasm were 4-fold significantly higher than those who had vasospasm (value of *p* = 0.047). (−)-S-nimodipine was significantly correlated with percentage MAP reduction. Similar results were obtained when the whole cohort was analyzed.

**Conclusion:**

The study was the first to investigate the potential association between nimodipine exposure following oral dosing and outcomes. In addition, it suggests differential effects of nimodipine enantiomers, shedding light on the potential utility of nimodipine enantiomers. Larger studies are needed.

## 1 Introduction

Aneurysmal subarachnoid hemorrhage (aSAH) is a critical neurological condition caused by the rupture of a cerebral blood vessel aneurysm, leading to bleeding into the subarachnoid space. Although aSAH accounts for 5% of all strokes, given the relatively young age at onset, it has a significant burden on patients’ productive life years. The average mortality rate for aSAH has been reported to range from 30 to 50%, leaving a significant proportion of survivors with disabilities (1–3). Neurological and medical complications following aSAH contribute significantly to the overall prognosis. The main complications secondary to aSAH significantly contributing to disability and unfavorable patient outcomes are delayed cerebral ischemia (DCI) and vasospasm. A substantial amount of research has been done with the aim of understanding the mechanisms of these complications and exploring potential therapeutic modalities for the sake of improving patient outcomes (4, 5). Several agents have been investigated to target vasospasm and DCI; however, nimodipine has been the only drug therapy that has been shown to significantly reduce the incidence of DCI secondary to aSAH and subsequently improves neurological outcomes post aSAH [relative risk 0.67 (95% CI 0.55–0.81)] (6–11). Therefore, nimodipine, a dihydropyridine calcium channel blocker with preferential effects on cerebral blood vessels, is currently considered a standard of care in aSAH management (Class I; Level of Evidence A) (2, 3).

American Heart Association (AHA) and Neurocritical Care Society guidelines recommend that all patients presenting with aSAH should receive a fixed dose of oral nimodipine 60 mg every 4 h for 21 days from aSAH onset (2, 3). Pharmacokinetic studies have reported extensive variability of nimodipine concentrations in various populations and in the setting of aSAH, with some patients having undetectable nimodipine plasma levels (12–17). The observed variability in nimodipine exposure may have been attributed to practice variations in nimodipine administration, systemic inflammation, disease severity, administration of concomitant interacting drugs, and cytochrome P450 polymorphism (17–22). While previous randomized controlled trials have found that nimodipine reduces the incidence of poor neurologic outcomes (defined by death, persistent vegetative state, and severe disability) by 40–86%, a significant proportion of patients in the nimodipine arm experienced poor outcomes (10, 23, 24). Therefore, it is not clear if all patients are getting the full benefit of nimodipine using a fixed-dose regimen. However, no prior studies have investigated whether an association exists between plasma nimodipine concentrations following oral dosing and clinical outcomes.

The aim of this pilot study was, hence, bi-faceted. The primary aim was to compare nimodipine systemic exposure in aSAH patients who experienced poor outcomes with those who experienced favorable outcomes at 90 days following aSAH. Poor outcomes were defined as modified Rankin Scale (mRS) score of 3–6, and favorable outcomes were defined as an mRS score of 0–2. Nimodipine systemic exposure was quantified using the area under the concentration-time curve at a steady state from 0 to 3 h (AUC_0-3h_) following a 60 mg oral dose. Furthermore, nimodipine maximum concentrations (C_max_) were compared as surrogate measures of AUC_0-3h_. The secondary aim was to identify covariates that are potentially associated with the observed nimodipine systemic exposure. This is through exploring trends in nimodipine concentrations categorized by different covariates. Since nimodipine is a chiral compound, we aimed to investigate the above comparisons for total nimodipine as well as both (+)-S and (−)-R enantiomers. To the best of our knowledge, this is the first study to report such comparisons.

## 2 Materials and methods

### 2.1 Study design

This was a pilot single-center prospective observational study. The study was approved by the Health Research Ethics Board of the University of Alberta, and informed consent was obtained from participants or from their substitute decision-makers (SDM). Patient recruitment occurred from June 2019 to February 2022.

### 2.2 Study population

Adult patients admitted to the Neuroscience Intensive Care Unit (ICU) at the University of Alberta Hospital and diagnosed with aSAH were included in the study. The inclusion criteria included aSAH patients aged 18–85 years who were receiving nimodipine 60 mg every 4 h either orally (PO) or through a feeding tube (FT) and presence of an intravascular catheter at the time of sampling to facilitate frequent blood withdrawals. The exclusion criteria were anticipated hospital length of stay of less than 48 h and non-aneurysmal SAH. Since our aims were exploratory and there was no similar work to this pilot study, we planned to enroll a convenient sample of 30 participants.

### 2.3 Data collection

Data were collected prospectively from the electronic health records following participants’ enrollment. Study data were managed using the REDCap (25, 26) electronic data capture tool hosted at the University of Alberta. The collected data included participants’ demographics [age, sex, height, weight, and body mass index (BMI)], pre-admission disability, history of hypertension, diabetes, chronic kidney disease, migraine, and liver disease (liver cirrhosis or Child Pugh class B or C). In addition, admission Glasgow coma score (GCS), Fisher scale, and World Federation of Neurological Surgeons (WFNS) grade were collected. Aneurysm location and treatment (e.g., endovascular coiling and surgical clipping) were also collected. The nimodipine administration record was also collected and included dose, frequency, duration, and route of administration (PO vs. FT). The administration and duration of liver microsomal enzyme (LME) inducing and inhibiting medications were recorded for the first 21 days of hospital stay or until discharge, whichever came first.

#### 2.3.1 Study outcomes

The primary clinical outcome collected was the mRS score at 90 days following aSAH. Participants’ mRS scores at 90 days were collected by contacting the participant or their SDM. The mRS is a measure of disability ranging from 0 (no symptoms at all) to 6 (dead), and it is the recommended functional outcome scale in clinical studies involving aSAH patients (27–29). A 90-day time period is the most common time frame for acute stroke trials (including aSAH) (30). Poor functional outcomes were defined as an mRS score of 3–6, and favorable outcomes were defined as an mRS score of 0–2.

Secondary clinical outcomes included delayed cerebral ischemia (DCI), vasospasm, and hospital mortality. DCI was defined according to Vergouwen et al. as “the occurrence of focal neurological impairment (such as hemiparesis, aphasia, apraxia, hemianopia, or neglect) or a decrease of at least 2 points on the Glasgow Coma Scale (either on the total score or on one of its individual components [eye, motor on either side, verbal]). This should last for at least 1 h, is not apparent immediately after aneurysm occlusion, and cannot be attributed to other causes by means of clinical assessment, CT or MRI scanning of the brain, and appropriate laboratory studies” (31). Vasospasm was defined as the presence of angiographic evidence of cerebral arterial narrowing (moderate to severe) as determined by the neuroradiologist utilizing digital subtraction angiography (DSA). Additional outcomes recorded included the length of stay in the hospital and ICU as well as the discharge disposition.

### 2.4 Study procedures

#### 2.4.1 Sample collection

Blood samples were collected at approximately one nimodipine 60 mg dose at a steady state. Since the reported half-life of nimodipine ranges from 1 to 2 h, and it generally takes 3 to 5 half-lives for a drug to achieve a steady state, we assumed that a steady state was reached after a minimum of 24 h of consistent dosing with a regimen of 60 mg every 4 h (17, 32). Blood samples (5 mL each) were collected at times 0 (just before the administration of nimodipine dose), 0.5, 1, 2, and 3 h following the administration of nimodipine dose. Samples were collected from an already established intravascular catheter (as part of the standard of care in our institution) by the bedside nurse in light-protected blood collection tubes (K2EDTA Vacutainer® lavender top, BD, San Jose, CA, USA). Samples were then sent to Alberta Precision Laboratories (APL) for processing and separation of plasma. Plasma samples were aliquoted into light-protected tubes and subsequently frozen at −70°C. Frozen samples were then transported to the principal investigator (S.H.M.) laboratory and stored at −80°C until analysis. To determine if nimodipine plasma concentrations were correlated with the intensity of blood pressure reduction, participants’ mean arterial pressure (MAP) values measured around the sampling nimodipine dose (as part of the standard of care) were also collected.

#### 2.4.2 Nimodipine plasma concentration determination

Nimodipine enantiomers [(−)-S and (+)-R] plasma concentrations were measured using an LC–MS/MS method validated at the principal investigator (S.H.M.) lab (33). Briefly, 300 μL of plasma samples or standard were added to 50 μL of the internal standard nifedipine (50 ng/mL), followed by alkalinization with 200 μL of 1 M sodium hydroxide. Aliquots of 4 mL of diethylether/hexane (1:1) were added, and the vortex was mixed for 5 min. The samples were then centrifuged at 2000 rpm for 10 min and frozen in a − 80 freezer for 20 min to separate the organic layer from the aqueous layer. Aliquots of the organic layer were transferred to clean tubes and evaporated to dryness under vacuum and no heating. Then, the residues were reconstituted in 125 μL of the mobile phase, and 40 μL were injected into the LC–MS/MS at a flow rate of 1 mL/min. The mobile phase consisted of methanol:water (75:25). The analysis was conducted using Shimadzu LC–MS/MS-8050 (Shimadzu Corporation, Kyoto, Japan) with a CBM-20A system controller, DGU-20A 5R degasser unit, SIL-30-AC autosampler, LC-30 AD binary pump, CTO-20 AC column oven, and LCMS-8050 triple quadrupole mass spectrometry detector. The chromatographic separation was carried out using an (S,S)-Whelk O1 (5 μm, 250 × 4.6 mm) chiral stationary phase column (Regis Technologies Inc., Morton Grove, IL, USA) with a KrudKatcher® Ultra guard column (Phenomenex, Torrance, CA, USA). LabSolutions® software version 5.91 (Shimadzu Kyoto, Japan) was utilized for data acquisition and chromatographic integration. The samples were run in singlets. During our assay development, running standard nimodipine samples consistently yielded equivalent peak ratios for the two enantiomers, indicating an equal quantity of each enantiomer in racemic nimodipine. The analytical method had intra- and inter-day coefficient of variation and percentage error within ±14%. Total nimodipine plasma concentrations were calculated by adding the values of (−)-S and (+)-R enantiomers concentrations at each time point. To eliminate bias, the analysts were blinded to the patients’ baseline characteristics.

#### 2.4.3 Plasma inflammatory markers determination

To determine whether markers of systemic inflammation are potentially associated with nimodipine exposure and patient outcomes, aliquots of the collected plasma samples were utilized to determine plasma cytokines by using commercially available ELISA kits (Invitrogen Co., Waltham, Massachusetts, United States). Cytokines included interleukin (IL-6), IL-1β, and tumor necrosis factor alpha (TNF-α), three markers potentially implicated with aSAH pathophysiology (34–36). The assays were conducted as per the manufacturer’s instructions. UV absorbance was measured using the Synergy H1 Hybrid Multi-Mode Plate Reader (BioTek Instruments, Inc., CA, United States).

### 2.5 Data analysis

Participants’ baseline characteristics, hospital course, outcomes, inflammatory markers, and nimodipine dosing were summarized. Continuous variables were presented as mean ± standard deviation (SD) if data were normally distributed and compared using the Student t-test. Alternatively, they were presented as median and interquartile range (IQR) if data were not normally distributed and compared using the Wilcoxon rank sum test. The Shapiro–Wilk test was utilized to assess the normality of continuous data. Categorical variables were presented as frequency and percentage n (%) and were compared using the χ^2^ or Fisher exact test, as appropriate. Nimodipine systemic exposure was quantified using the area under the concentration-time curve at a steady state from 0 to 3 h (AUC_0-3h_) following a 60 mg oral dose. Individual AUC_0-3h_ were calculated from nimodipine concentration-time data using the linear trapezoidal method utilizing PKanalix® software version 2021R1 (Lixoft, Antony, France). Furthermore, nimodipine maximum (C_max_) and minimum (C_min_) concentrations were determined from nimodipine concentration-time data. The correlations of MAP percentage drop, calculated as the difference in pre-dose MAP and the lowest MAP value within 2 h of nimodipine administration divided by pre-dose MAP multiplied by 100, with total, (−)-S and (+)-R nimodipine C_max_ were determined using Pearson’s correlation coefficient. Median AUC_0-3h_ and C_max_ were compared between those with poor outcomes (mRS of 3–6) and those with favorable outcomes (mRS of 0–2) using the Wilcoxon rank sum test. To check if nimodipine enantiomers have differential effects, the above comparisons were also conducted for both (−)-S and (+)-R enantiomers. The contribution of various covariates on nimodipine exposure was determined using categorization. Explored covariates were age, BMI, presence of interacting drugs, liver disease, WFNS grade, inflammatory mediators, and nimodipine route of administration. Missing data, if any, were handled by complete case analysis. A value of *p* of <0.05 was considered statistically significant. Data analysis was conducted using STATA software version 15 (STATA Corporation, College Station, TX, USA).

## 3 Results

A total of 31 participants were recruited. One participant had their intravascular catheter removed and was sent to the ward just after enrollment. Therefore, 30 participants were included in the current study. Table 1 depicts the baseline characteristics of the included participants. Females comprised 60% of the study participants, and the mean age of the cohort was 57 ± 12.1 years. All participants were without baseline disability (i.e., preadmission mRS of 0). Forty percent of the participants presented with poor grade aSAH (WFNS of 3-5). Two-thirds of the ruptured aneurysms were secured by endovascular coiling (63.3%), while the remaining 36.7% were secured by surgical clipping. All participants received nimodipine treatment within the first 24 h of hospital admission with a median (IQR) duration of 14.5 (12–20) days. A summary of nimodipine dosing in the current study is shown in Table 2.

**Table 1:** Baseline characteristics of the enrolled participants.

Characteristic	All cohort (*n* = 30)	mRS 90d 0-2 (*n* = 20)	mRS 90d 3-6 (*n* = 9)	Value of *p*
Age (years)	57 ± 12.1	55.5 ± 9.9	64.3 ± 8.2	**0.027**
Females	18 (60)	12 (60)	5 (55.6)	1.0
Height (cm)	172.4 ± 11.8	172.5 ± 8.9	172.5 ± 17.6	0.998
Weight (kg)	81.2 ± 22.9	80.6 ± 21.2	85.3 ± 27	0.619
BMI	27.2 ± 6.7	27 ± 6.6	28.3 ± 6.9	0.645
Hypertension	11 (36.67)	5 (25)	6 (66.67)	**0.048**
Delayed presentation	5 (16.67)	5 (25)	0 (0)	0.153
Aneurysm location				**0.034**
MCA	6 (20)	6 (30)	0 (0)	
ACOM	8 (26.67)	3 (15)	4 (44.44)	
PCOM	6 (20)	5 (25)	1 (11.11)	
BASILAR	2 (6.67)	0 (0)	2 (22.22)	
OTHER LOCATION	8 (26.67)	6 (30)	2 (22.22)	
Aneurysm treatment				0.237
Coiling	19 (63.33)	14 (70)	4 (44.44)	
Clipping	11 (36.67)	6 (30)	5 (55.56)	
Fisher Scale				0.082
2	8 (26.67)	7 (35)	0 (0)	
3	4 (13.33)	3 (15)	1 (11.11)	
4	18 (60)	10 (50)	8 (88.9)	
Admission GCS	14 (14–15)	14.5(14–15)	14 (12–14)	**0.015**
WFNS grade				0.130
1	11 (36.67)	10 (50)	1 (11.11)	
2	7 (23.33)	4 (20)	2 (22.22)	
3	8 (26.67)	5 (25)	3 (33.33)	
4	3 (10)	1 (5)	2 (22.22)	
5	1 (3.33)	0 (0)	1 (11.11)	
Poor WFNS (grades 3–5)	12 (40%)	6 (30)	6 (66.67)	0.106

Categorical variables are presented as *n* (%), while continuous variables are presented as mean ± standard deviation. Admission GCS is presented as median (interquartile range). Those who had favorable (0–2) modified Rankin scale at 90 days (mRS 90d) and poor (3-6) mRS 90d were compared. None of the patients had chronic kidney disease, acute kidney injury, or liver disease. One patient had diabetes type II and was in the poor mRS group. All patients had a baseline mRS of 0. Delayed presentation was defined as presentation to the hospital > 96 h from symptom onset. ACOM, anterior communicating artery; GCS, Glasgow Coma Score; MCA, middle cerebral artery; PCOM, posterior communicating artery; WFNS, World Federation of Neurological Surgeons. WFNS grades of 3 to 5, we considered poor (high) grade; otherwise, they are considered low grade. One participant was lost to follow-up and thus was excluded from mRS comparisons. Bold values, statistically significant.

**Table 2:** Nimodipine dosing history in the enrolled participants.

Characteristic	All cohort (*n* = 30)	mRS 90d 0-2 (*n* = 20)	mRS 90d 3-6 (*n* = 9)	Value of *p*
Number of days of nimodipine	14.5 (12–20)	13.5 (11.5–19.5)	20 (14–21)	0.306
Days on nimodipine 60 mg PO	12.5 (9–18)	13.5 (10.5–18.5)	5 (0–17)	0.09
Days on nimodipine 30 mg PO	0 (0–0)	0 (0–0)	0 (0–0)	0.966
Days on nimodipine 60 mg FT	0 (0–3)	0 (0–0)	5 (0–13)	**0.004**
Days on nimodipine 30 mg FT	0 (0–0)	0 (0–0)	0 (0–1)	0.26
Nimodipine exposure (%)	100 (95–100)	100 (100–100)	100 (95–100)	0.451

Data are presented as median (interquartile range). Those who had favorable (0-2) modified Rankin scale at 90 days (mRS 90d) and poor (3-6) mRS 90d were compared. Nimodipine exposure was calculated as a percentage: the ratio of number of days of nimodipine divided by the hospital length of stay or 21 days, whichever shorter multiplied by 100. FT, administered via feeding tube; PO, administered orally. One participant was lost to follow up and thus was excluded from mRS comparisons. Bold values, statistically significant.

### 3.1 Study outcomes

All participants had their 90-day mRS recorded except one patient was lost to follow-up. Twenty participants (69%) had favorable outcomes (mRS of 0–2) and 9 (31%) had poor outcomes (mRS of 3-6). Comparison of the baseline characteristics and nimodipine dosing of those who had poor outcomes to those who had favorable outcomes are depicted in Tables 1, 2, respectively. Compared to the favorable outcome group, participants’ who had poor outcomes were older (64.3 ± 8.2 vs. 55.5 ± 9.9 years, value of *p* 0.027), had higher prevalence of hypertension, and had lower admission GCS. In terms of nimodipine treatment course completion, the ratio of the number of days of nimodipine treatment divided by the hospital length of stay or 21 days, whichever is shorter was multiplied by 100, and both groups had similar median treatment course percentages (100%) (Table 2).

All participants but one had DSA conducted, where 15 participants (50%) had angiographic evidence of vasospasm and 8 (26.7%) developed DCI. One patient did not undergo angiography, but transcranial Doppler (TCD) showed severe vasospasm and CT had multiple infarcts and therefore was coded to have VSP and DCI. A total of 20 (66.7%) participants had mechanical ventilation with a median (IQR) duration of 1 (0–3) day for the whole cohort. A total of 18 (60%) participants had hydrocephalus and had an external ventricular drain (EVD) for a median (IQR) duration of 10.5 (0–14) days for the whole cohort. Median (IQR) ICU and hospital stays were 13.5 (8–18) and 18 (12–24) days, respectively.

### 3.2 Nimodipine plasma concentrations

**Figure 1:**
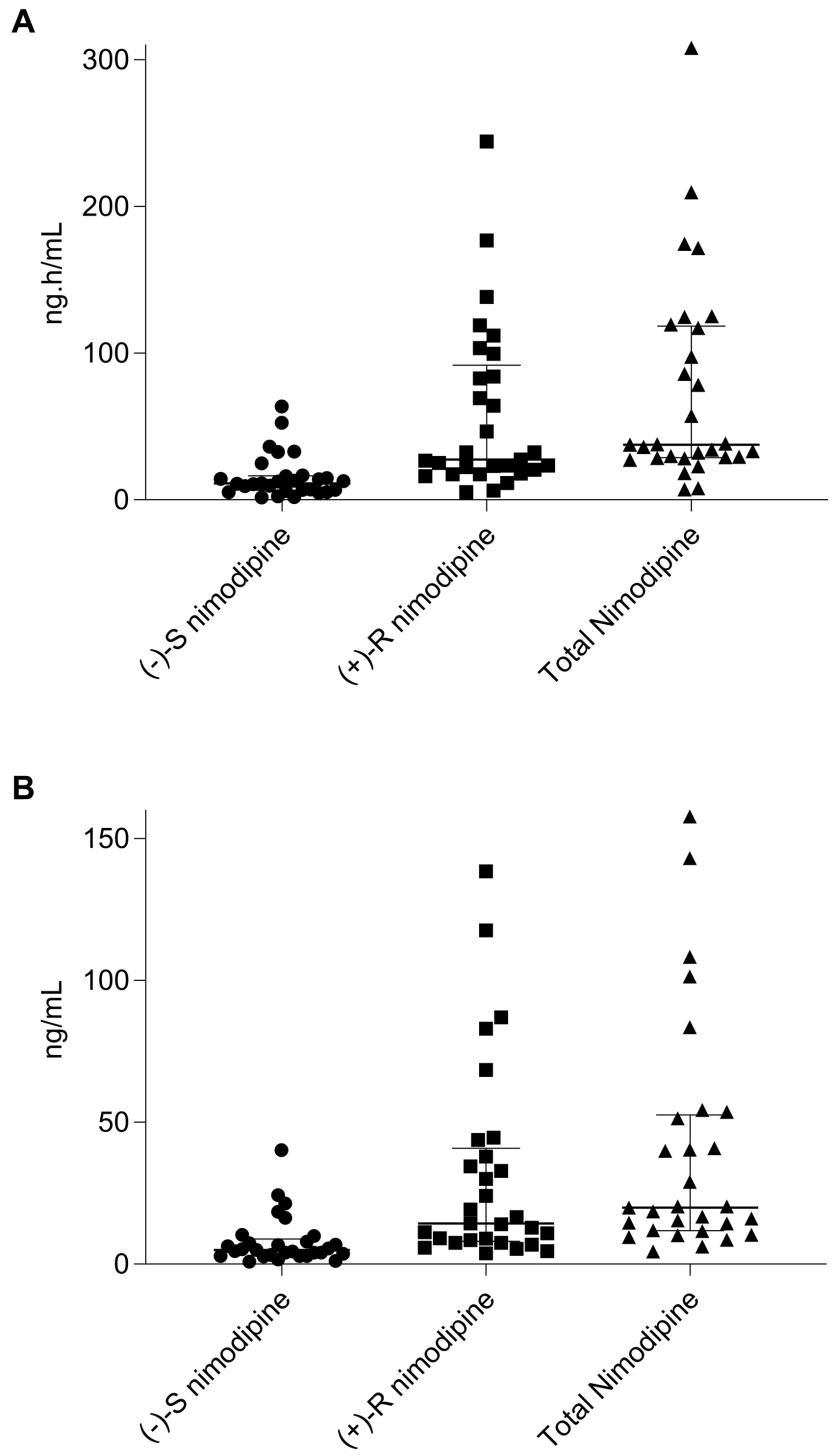
Scatter plot and median (interquartile range) of **(A)**, nimodipine area under the concentration-time curve (AUC_0-3h_) from 0 to 3 h for (−)-S, (+)-R, and total nimodipine; **(B)**, nimodipine maximum concentrations (C_max_) for (−)-S, (+)-R, and total nimodipine. *n* = 29 (one participant who only had two samples collected and was consequently excluded from the pharmacokinetic calculations).

All participants had five blood samples collected around a 60 mg dose at the steady state, with the exception of one participant who only had two samples collected and was consequently excluded from the pharmacokinetic calculations. The sampling days ranged from 2 to 11 days following nimodipine initiation, with a median (IQR) of 4 (3–6) days. Six (20%) participants were receiving nimodipine via the feeding tube and others orally. A large variability in total, (−)-S and (+)-R nimodipine enantiomer concentrations was observed (Table 3; Figure 1). (−)-S-nimodipine plasma concentrations were approximately 3-fold lower than the (+)-R enantiomer. Both C_max_ and C_min_ for total nimodipine were strongly correlated with AUC_0-3h_ (*r* = 0.92, value of *p* < 0.001 for C_max_ vs. AUC_0-3h_; *r* = 0.97, value of *p* < 0.001 for C_min_ vs. AUC_0-3h_), suggesting that C_max_ and C_min_ could be utilized as surrogate measured of AUC_0-3h_. Similar associations were present for both (−)-S and (+)-R enantiomers.

**Table 3:** Nimodipine pharmacokinetic parameters.

Parameter*	(−)-S nimodipine			(+)-R nimodipine			Total nimodipine		
	Median (IQR)	Min	Max	Median (IQR)	Min	Max	Median (IQR)	Min	Max
C_min_ (ng/mL)	3 (1–5)	0.25	14.8	7 (5–18)	0.82	66.8	9 (8–22)	1.1	81.6
C_max_ (ng/mL)	5 (3–8)	0.89	40.2	14 (9–38)	3.7	138.5	20 (12–51)	4.5	157.8
T_max_ (h)	0.7 (0.5–1)	0	3.03	0.5 (0.5–1)	0	2	0.7 (0.5–1)	0	3.58
AUC_0-3h_ (ng.h/mL)	11 (7–16)	1.63	63.72	28 (21–84)	5.17	244.23	38 (29–117)	7.23	307.94

AUC_0-3h_, area under the concentration-time curve from 0 to 3 h; C_max_, nimodipine maximum concentration; C_min_, nimodipine trough concentration; T_max_, time to maximum drug concentration; *, *n* = 29 (one participant who only had two samples collected and was consequently excluded from the pharmacokinetic calculations).

### 3.3 Nimodipine exposure and patient outcomes

**Figure 2:**
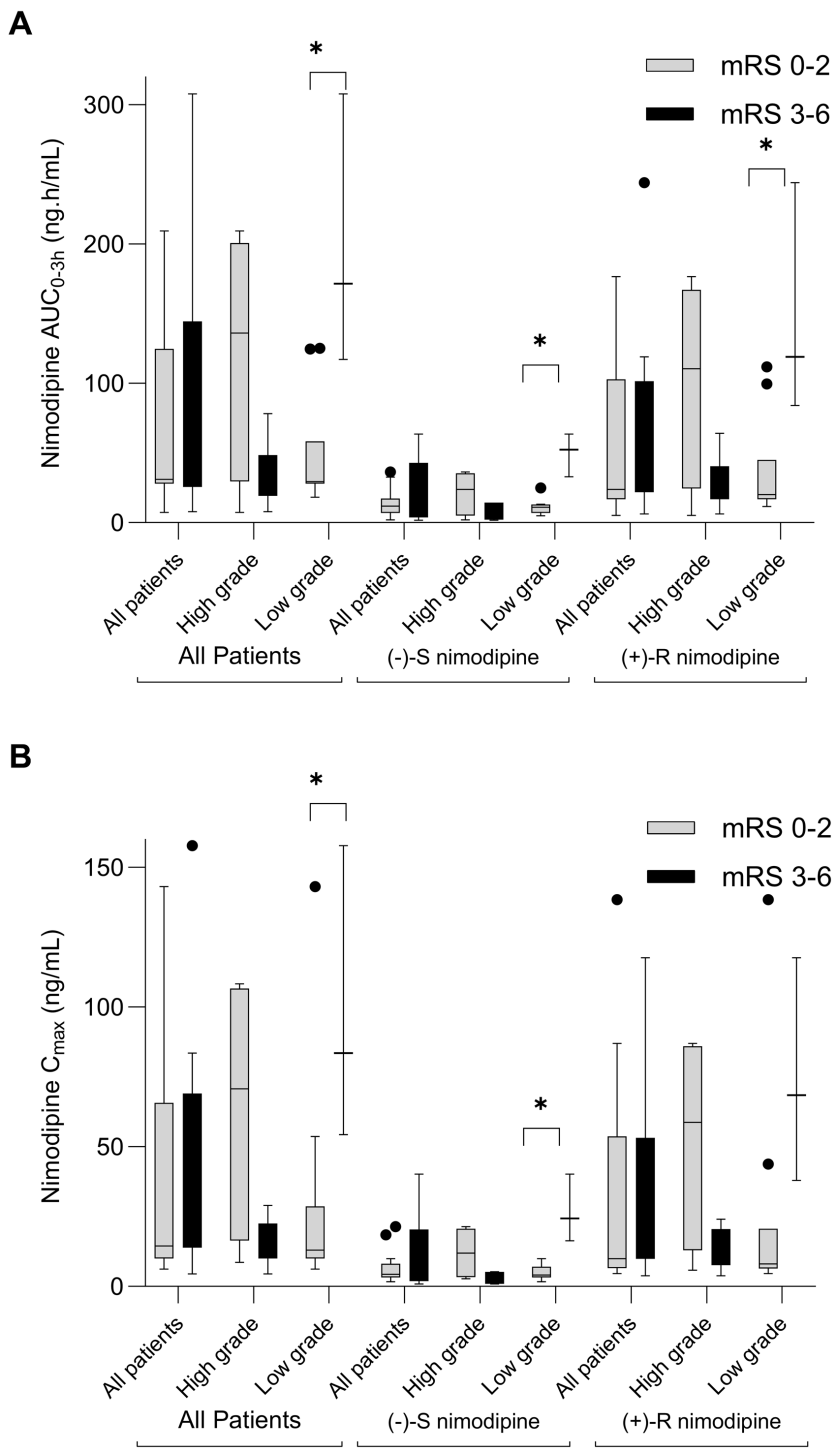
Box-plot comparison of nimodipine exposure in aSAH participants who experienced poor outcomes (modified Rankin scale, mRS 3-6) to those who experienced favorable outcomes (mRS 0-2) at 90 days following aSAH. **(A)** AUC_0-3h_ comparison; **(B)** C_max_ comparison. High grade, World Federation of Neurological Surgeons (WFNS) grade of 3-5; Low Grade, WFNS of 1-2. *n* = 23 (exclusions: 1 lost to follow-up, 1 did not have sufficient samples, and 5 with delayed presentation). *p < 0.05.

Nimodipine systemic exposure in aSAH participants who experienced poor outcomes (mRS 3–6) was compared to those who experienced favorable outcomes (mRS 0–2) at 90 days following aSAH. Five participants had delayed presentation exceeding 96 h (time window for nimodipine administration); thus, they were excluded from the comparison. This is because aSAH patients who present with a delay may have different characteristics and outcomes compared to those with immediate or early presentation (37–39). Figure 2 depicts the comparisons of nimodipine AUC_0-3h_ and C_max_ by mRS at 90 days. As seen in the figure, AUC_0-3h_ and C_max_ were not significantly different when the whole cohort was compared. However, when stratified by the admission WFNS grade, a split occurred. Among those who presented with high grade (WFNS 3–5), nimodipine AUC_0-3h_ in those with favorable outcomes were 4-fold higher than in those with poor outcomes [136(52–192) vs. 33(23–39) ng.h/mL, respectively, value of *p* = 0.2]. Similarly, nimodipine C_max_ in those was favorable outcomes was higher than in those with poor outcomes [71(24–105) vs. 18 (12-20) ng/mL, respectively, value of *p* = 0.14]. Similar findings were observed when individual nimodipine enantiomers were analyzed (Figure 2; Supplementary Tables 1, 2). On the other hand, among those presented with a low grade (WFNS 1–2), nimodipine AUC_0-3h_ in those with favorable outcomes was significantly lower than in those with poor outcomes [30(28–36) vs. 172(117–308) ng.h/mL, respectively, value of *p* = 0.03]. Similarly, nimodipine C_max_ in those was favorable outcomes was significantly lower than in those with poor outcomes [13(10–20) vs. 84(54–158) ng/mL, respectively, value of *p* = 0.03]. Similar findings were observed when individual nimodipine enantiomers were analyzed (Figure 2; Supplementary Tables 1, 2).

Furthermore, similar results were obtained when the whole cohort, with AUC_0-3h_, C_max_, and follow-up data, was analyzed (*n* = 28). AUC_0-3h_ and C_max_ were not significantly different when the whole cohort was compared [AUC_0-3h_ (favorable outcome: 34 (29–120) vs. poor outcome: 39 (29–117) ng.h/mL, value of *p* = 0.71); C_max_ (favorable outcome: 17 (10–51) vs. poor outcome: 20 (16–54) ng/mL, value of *p* = 0.45)]. When stratified by admission WFNS grade, a split occurred. Among those presented with high grade (WFNS 3–5), nimodipine AUC_0-3h_ in those with favorable outcomes was higher than in those with poor outcomes [120(98–175) vs. 33 (23–39) ng.h/mL, respectively, value of *p* = 0.1]. Similarly, nimodipine C_max_ in those with favorable outcomes was significantly higher than in those with poor outcomes [51(40–101) vs. 18 (12–20) ng/mL, respectively, value of *p* = 0.07]. Similar findings were observed when individual nimodipine enantiomers were analyzed. On the other hand, among those presented with a low grade (WFNS 1–2), nimodipine AUC_0-3h_ in those with favorable outcomes was significantly lower than in those with poor outcomes [33(29–38) vs. 172(117–308) ng.h/mL, respectively, value of *p* = 0.02]. Similarly, nimodipine C_max_ in those with favorable outcomes was significantly lower than in those with poor outcomes [15 (10–20) vs. 84(54–158) ng/mL, respectively, value of *p* = 0.02]. Similar findings were observed when individual nimodipine enantiomers were analyzed.

Nimodipine exposure was also compared between individuals who experienced vasospasm and those who did not (Supplementary Tables 1, 2). All of the comparisons were not statistically different, except for (−)-R and total nimodipine among the participants who presented with high grades. (+)-R nimodipine AUC_0-3h_ in those who did not develop vasospasm were 4-fold significantly higher than those who had vasospasm [83(64–138) vs. 23 (6–24) ng.h/mL, respectively, value of *p* = 0.047]. Similarly, (−)-R nimodipine C_max_ in those who did not develop vasospasm were 4-fold significantly higher than those who had vasospasm [34(24-83) vs. 9 (6–11) ng/mL, respectively, value of *p* = 0.009]. With regard to DCI, trends of differences in nimodipine exposure were observed, but none reached statistical significance (Supplementary Tables 1, 2). Similar results were obtained when the whole cohort was analyzed.

### 3.4 Nimodipine exposure and MAP reduction

**Figure 3:**
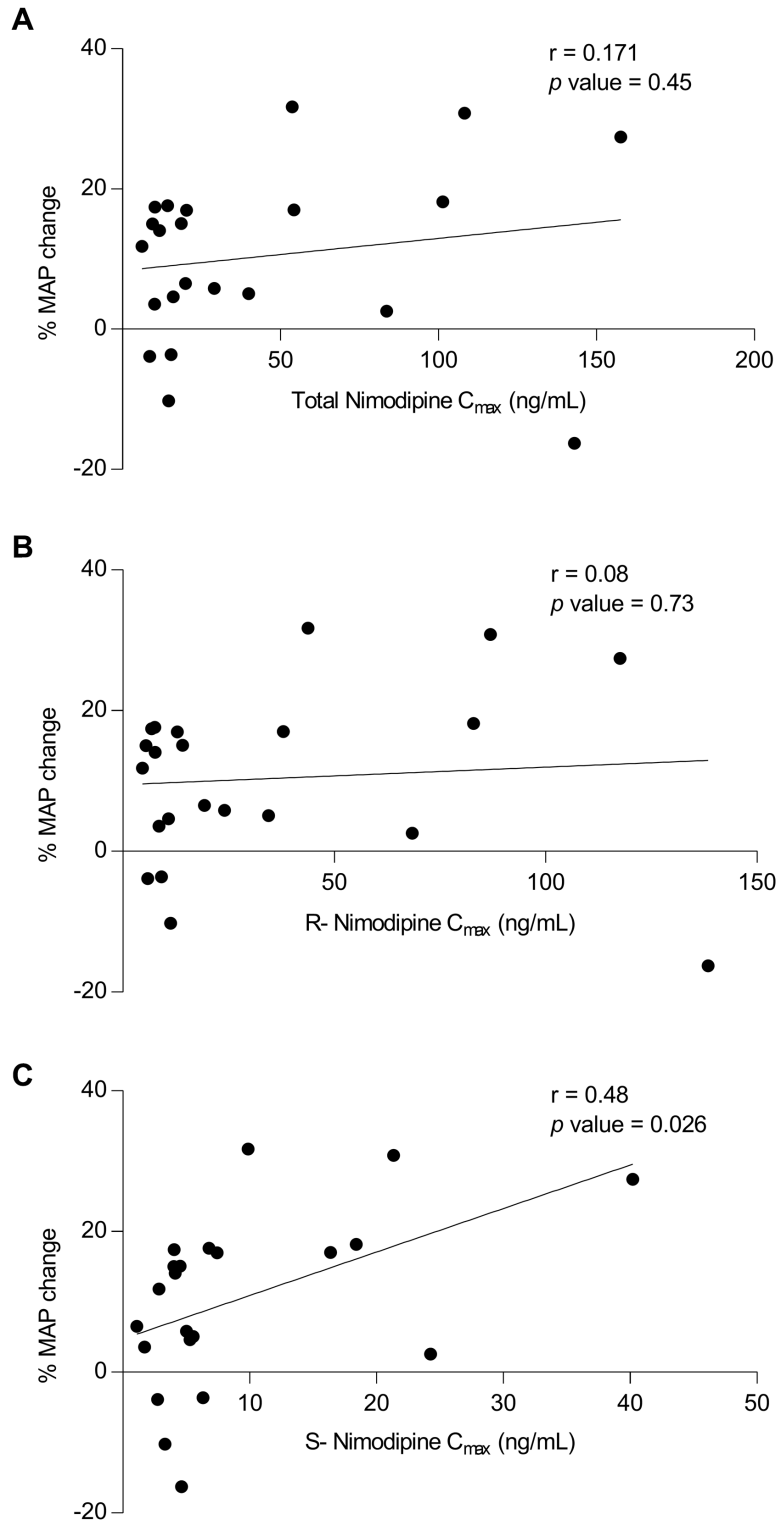
Plot of nimodipine maximum concentrations (C_max_) vs. the percentage mean arterial pressure (MAP) changes following nimodipine administration. **(A)** Total nimodipine; **(B)** (+)-R-enantiomer; **(C)** (−)-S-enantiomer. *n* = 22 included (Excluded participants: 2 were on vasopressors, 2 received intermittent intravenous labetalol concomitantly, 3 did not have MAP data, and 1 did not have C_max_ value).

To determine whether nimodipine enantiomers exhibit differential effects on blood pressure, where hypotension is the main intolerance factor with nimodipine administration, MAP values measured around the sampling nimodipine dose were collected. To avoid the influence of concomitant therapies potentially confounding MAP measurements, those who were concomitantly treated with antihypertensives (*n* = 2, intermittent IV labetalol) and vasopressors (*n* = 2, milrinone and norepinephrine) were excluded from the analysis. Three participants had no MAP data, and one participant did not have a C_max_ value, and therefore, they were excluded. As depicted in Figure 3, there was a significant correlation observed between (−)-S nimodipine alone and the percentage reduction in MAP.

### 3.5 Plasma inflammatory markers

**Figure 4:**
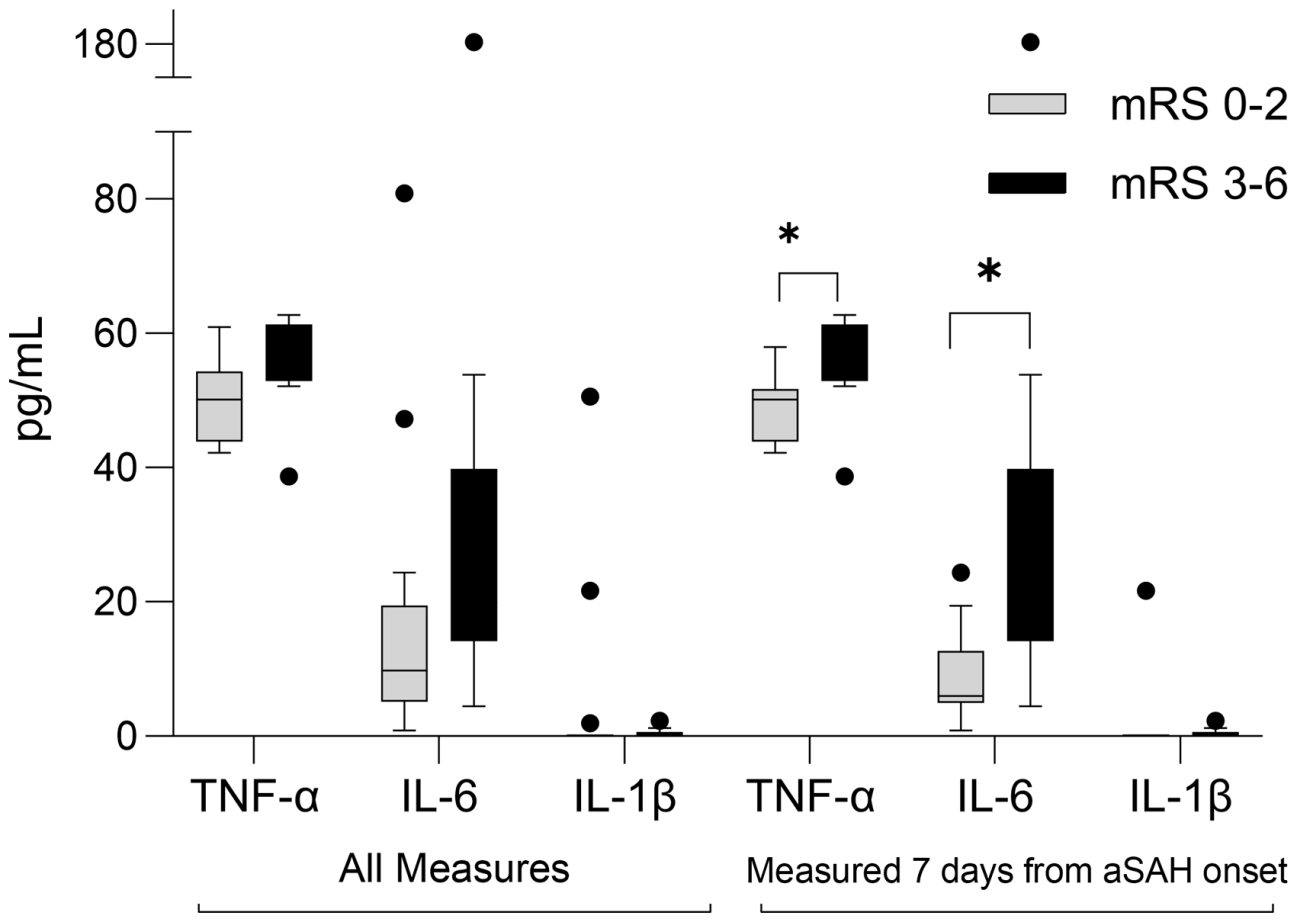
Box-plot comparison of inflammatory mediators in aSAH participants who experienced poor outcomes (modified Rankin scale, mRS 3-6) to those who experienced favorable outcomes (mRS 0-2) at 90 days following SAH. All measures (*n* = 24, exclusions: 1 lost to follow-up, 5 delayed presentation); Measured 7-days from aSAH onset (*n* = 20). *p < 0.05 IL, interleukin, TNF, tumor necrosis factor.

Three cytokine plasma levels were measured: IL-6, IL-1β, and TNF-α, three markers potentially implicated with aSAH pathophysiology. Figure 4 summarizes inflammatory mediators’ levels among those with favorable and poor mRS outcomes. Excluding those with delayed presentation, plasma TNF-α and IL-6 measured in the first 7 days from SAH onset were significantly elevated in participants with poor mRS at 90 days [TNF-α: 55(54–60) vs. 50(44–52) pg./mL, respectively, value of *p* = 0.03; IL-6: 20(18–26) vs. 6(5–13) pg./mL, respectively, value of *p* = 0.02].

### 3.6 Covariates potentially associated with nimodipine plasma concentrations

To determine potential covariates associated with nimodipine systemic exposure (AUC_0-3h_), we explored trends in nimodipine concentrations categorized by different covariates. The explored covariates were age, sex, BMI, WFNS grade, co-administration of phenytoin, inflammatory markers, and nimodipine route of administration (Table 4). Participants with plasma TNF-α levels above 57 pg./mL and those with detectable IL-1β trended toward elevated nimodipine AUC_0-3h_. Furthermore, there was a trend toward lower nimodipine AUC in those receiving nimodipine enterally via the feeding tube and those concomitantly treated with phenytoin (an LME inducer).

**Table 4:** Nimodipine systemic exposure categorized by various covariates.

Covariate		Total AUC_0-3h_ (ng.h/L)	(−)-S AUC_0-3h_ (ng.h/L)	(+)-R AUC_0-3h_ (ng.h/L)
Age (years)	<60 (*n* = 15)	37 28–125)	11 (6–15)	32 (17–112)
	≥60 (*n* = 14)	37 (29–117)	14 (7–17)	27 (23–84)
Sex	Males (*n* = 12)	33 (28–107)	9 (6–20)	26 (20–84)
	Females (*n* = 17)	38 (29–120)	13 (9–16)	33 (21–103)
BMI (kg/m^2^)	<18.5 (*n* = 2)	35 (32–38)	6 (5–7)	29 (25–33)
	18.5 - < 25 (*n* = 11)	29 (27–120)	11 (5–16)	23 (18–103)
	25 - < 30 (*n* = 6)	58 (33–98)	12 (6–15)	48 (23–83)
	≥30 (*n* = 10)	62 (30–125)	14 (11–33)	48 (17–112)
Phenytoin co-administration	Phenytoin (*n* = 4)	33 (26–68)	10 (4–15)	23 (22–53)
	No Phenytoin (*n* = 25)	38 (29–120)	11 (7–17)	32 (18–100)
WFNS grade	High grade (*n* = 11)	39 (23–120)	14 (2–16)	32 (21–103)
	Low grade (*n* = 18)	35 (29–117)	11 (7–16)	27 (18–84)
Route of administration	Oral (*n* = 23)	38 (29–120)	11 (7–17)	32 (18–100)
	Enteral via FT (*n* = 6)	33 (23–78)	10 (2–14)	23 (21–64)
TNF-α (pg/mL)	<45 (*n* = 8)	36 (23–91)	8 (6–18)	28 (17–73)
	45–52 (*n* = 7)	34 (29–120)	13 (11–16)	28 (17–103)
	53–57 (*n* = 7)	36 (29–78)	9 (5–14)	26 (23–64)
	>57 (*n* = 7)	86 (28–210)	17 (53–5)	69 (21–177)
IL-6 (pg/mL)	<6.0 (*n* = 8)	33 (29–106)	12 (10–15)	24 (18–91)
	6.0–15 (*n* = 7)	38 (27–98)	13 (5–15)	33 (17–83)
	16–30 (*n* = 7)	29 (8–125)	5 (2–33)	23 (6–100)
	>30 (*n* = 7)	39 (36–120)	11 (7–16)	32 (27–103)
IL-1β (pg/mL)	0 (*n* = 23)	34 (28–86)	11 (5–14)	25 (17–69)
	>0 (*n* = 6)	107 (39–120)	16 (10–33)	84 (32–103)

Data are presented as median (interquartile range). AUC_0-3h_, area under the concentration-time curve from 0 to 3 h; BMI, body mass index, IL, interleukin; TNF, tumor necrosis factor; WFNS, World Federation of Neurological Surgeons. High grade, World Federation of Neurological Surgeons (WFNS) grade of 3-5; Low Grade, WFNS of 1-2. Cytokine ranges represent quantiles that were generated using STATA. *n* = 29 (one participant who only had two samples collected and was consequently excluded from the pharmacokinetic calculations).

## 4 Discussion

This was a pilot single-center prospective observational study in adult patients admitted to the University of Alberta Hospital with aSAH. The study is the first to investigate the potential association between nimodipine exposure following oral dosing and patient outcomes. Furthermore, it is the first study to suggest differential effects of individual nimodipine enantiomers, shedding light on the potential utility of individual nimodipine enantiomers.

In the present study, a large variability in nimodipine concentrations was observed (Table 3). Pharmacokinetic studies have reported considerable variability in nimodipine concentrations among aSAH patients, with some individuals showing undetectable levels in their plasma (12–17). For instance, Soppi et al. conducted a study to analyze nimodipine concentrations in patients with aSAH who followed the standard 60 mg oral dose in every 4 h regimen (12). They found that C_max_ was as low as 1 ng/mL to 57 ng/mL in individuals taking tablets, while those receiving an oral suspension exhibited concentrations of 0.9–1.7 ng/mL. Similarly, Abboud et al. compared plasma concentrations of nimodipine between patients who received it parenterally and those who received it orally (16) Among patients who ingested whole nimodipine tablets, the AUC was almost double compared to those who received it through an enteral feeding tube (16). Additionally, two patients with severe SAH exhibited undetectable nimodipine concentrations. The observed variability in nimodipine exposure may be secondary to practice variations in nimodipine administration, systemic inflammation, disease severity, administration of concomitant interacting drugs, and cytochrome P450 polymorphism (17–21). Nevertheless, it is not clear if minimal or lack of systemic exposure to oral nimodipine attenuates the benefit and contributes to worsening patient outcomes.

Following the exclusion of those with delayed presentation, nimodipine AUC_0-3h_ in participants who experienced poor outcomes (mRS 3-6) was compared to those who experienced favorable outcomes (mRS 0-2) at 90 days following SAH. Among those who presented with high disease severity, nimodipine AUC_0-3h_ in those with favorable outcomes was 4-fold higher than in those with poor outcomes. On the other hand, among those presented with a low grade (WFNS 1-2), nimodipine AUC_0-3h_ in those with favorable outcomes was significantly lower than in those with poor outcomes. Similar results were obtained when the whole cohort with follow-up data was analyzed. Although such a finding could be attributed to the small sample size of the study and non-adjustment for confounders, it may suggest the potential increased benefit of nimodipine in those with increased disease severity. A larger multicenter study is needed to determine if such associations persist after controlling for confounders. No previous studies have addressed if an association exists between plasma nimodipine concentrations after oral dosing and clinical outcomes. The study conducted by Riva et al. suggested a link between nimodipine concentrations in cerebrospinal fluid (CSF) and neurological outcomes after 9 months from the onset of aSAH in a group of 23 patients (40). However, they were unable to establish a similar correlation with plasma concentrations. It is worth noting that all patients who received nimodipine through intravenous infusion, resulted in plasma concentrations equal to or greater than 25 ng/mL, which surpasses levels observed in certain patients who received oral doses. Furthermore, determining the association between nimodipine plasma concentration and outcomes might be of greater value than determining CSF concentrations. This is because measuring CSF samples is not practical as not all patients will have readily available CSF.

Nimodipine is a dihydropyridine calcium channel blocker with a chiral carbon atom. The currently marketed compound is a racemic mixture of (+)-R and (−)-S nimodipine. Our study suggested differential effects of individual nimodipine enantiomers, shedding light on the potential utility of individual nimodipine enantiomers. We found that that among those who presented with severe disease, AUC_0-3h_ of (+)-R nimodipine in those who did not develop vasospasm was 4-fold significantly higher than in those who had vasospasm. Such a difference was not apparent with (−)-S nimodipine. On the other hand, our data suggested that (−)-S but not (+)-R nimodipine concentrations were correlated with percent MAP reduction (*r* = 0.48, value of *p* = 0.03), and the (−)-S enantiomer could be the culprit for the observed nimodipine-induced hypotension, which is the main challenge limiting the dosing of nimodipine (41). Towart et al. discovered that (−)-S nimodipine exhibits approximately double the potency as a vasorelaxant compared to the racemic mixture, supporting our finding of such differential pharmacology (42). In addition, in our study, (−)-S nimodipine plasma concentrations were approximately 4-fold lower than the (+)-R enantiomer. Enantioselective first-pass metabolism leads to a more rapid elimination of the (−)-S enantiomer compared to the (+)-R enantiomer after oral administration (20, 43–45). However, such differential effects were not obvious when nimodipine was given intravenously, resulting in higher concentrations of the (−)-S enantiomer compared to oral dosing. This could potentially explain, at least in part, the excessive hypotensive effect reported following the IV route (46). These findings, although preliminary, are hypothesis generating, suggesting that using (+)-R nimodipine instead of the racemic mixture could potentially retain the benefits of racemic nimodipine while reducing its hypotensive effect, a main limiting factor of nimodipine therapy. Hypotension should be avoided to avoid cerebral hypoperfusion and complications following aSAH (2, 46). Further studies are needed to investigate this hypothesis.

In order to identify potential covariates associated with nimodipine systemic exposure, we examined trends in nimodipine concentrations categorized by various covariates (Table 4). Participants with plasma TNF-α levels above 57 pg./mL and those with detectable IL-1β tended toward elevated nimodipine exposure. This was similar to the findings of an earlier study conducted by S.H.M. where systemic inflammation resulted in significantly increased concentrations of the calcium channel blocker (CCB) verapamil (47–49). This has been attributed to inflammation-induced downregulation of cytochrome P450 enzymes and increased verapamil protein binding (47, 49–51). However, despite the increased concentrations, verapamil’s pharmacological effect was compromised secondary to inflammation-induced downregulation of L-type calcium channels, verapamil’s target receptor (47, 49, 52). Similarly, the pharmacological response to nifedipine was also compromised in the setting of inflammation (53, 54). It is not known, however, if these inflammation-induced alterations result in reduced nimodipine effects as seen with other CCBs. In the present study, plasma TNF-α and IL-6 measured in the first 7 days from SAH onset were elevated in participants with poor mRS at 90 days. This was similar to several articles reporting an association between increased systemic inflammation and poor outcomes following aSAH (34–36, 55, 56). However, none investigated if the observed poor outcomes could be attributed, at least in part, due to altered nimodipine actions and disposition. Further studies are needed.

Furthermore, there was a trend toward lower nimodipine AUC_0-3h_ in those receiving nimodipine enterally via the feeding tube and those concomitantly treated with phenytoin (an LME inducer). We previously reported an association between the nimodipine administration technique and patient outcomes where patients receiving crushed nimodipine tablets enterally had worse outcomes compared to those who received whole tablets after controlling for disease severity (57). We confirmed such findings in a multicenter retrospective study where we compared various enteral administration formulations and techniques (22). This could be attributed to the reduced oral bioavailability of enteral nimodipine, especially the manufacturer recommends against tablet crushing due to the risk of reduced absorption (58).

Our study was limited by the small sample size. We were unable to control confounders associated with the tested outcomes in multivariate analysis. Moreover, it is crucial to exercise caution when interpreting our results primarily due to the presence of contradictory findings related to our primary research question. This situation heightens the risk of confirmation bias influencing our conclusions. Although the study was not powered to detect differences, it was meant to identify trends that will potentially be utilized to design a larger multicenter study.

## 5 Conclusion

aSAH is a severe medical crisis requiring urgent medical and surgical attention. The demonstrated advantages of administering nimodipine in aSAH underscore the importance of providing it to all patients, as long as it is well tolerated. An individualized approach to the administration of nimodipine remains an area of ongoing research as the association between variations in pharmacokinetic parameters and clinical outcomes remains unclear. The findings of this research aimed to pave the way for further research to determine whether nimodipine exposure is an independent predictor of aSAH outcomes and ways to optimize the dosing of nimodipine in aSAH to possibly increase the likelihood of achieving pharmacokinetic measures associated with treatment success and improved patients’ clinical outcomes.

## Data availability statement

The raw data supporting the conclusions of this article will be made available by the authors, without undue reservation.

## Ethics statement

The studies involving humans were approved by the Health Research Ethics Board of the University of Alberta. The studies were conducted in accordance with the local legislation and institutional requirements. The participants provided their written informed consent to participate in this study.

## Author contributions

SM: conceptualization, supervision, project administration, study design, statistical analysis, writing original draft, manuscript revision, and data interpretation. FH: data acquisition, writing review, and data interpretation. FI: data acquisition, writing review, and data interpretation. SF: data acquisition, writing review, and data interpretation. SL: data acquisition and writing review. CO’K: study design and writing review. JK: study design and writing review. All authors contributed to the article and approved the submitted version.
