# Supplementary material for: Nimodipine systemic exposure and outcomes following aneurysmal subarachnoid hemorrhage: a pilot prospective observational study (ASH-1 study)
Source: Front Neurol. 2024 Jan 5;14:1233267. doi: 10.3389/fneur.2023.1233267 (PMC10796587; doi:10.3389/fneur.2023.1233267)
Supplement: Supplementary file 1 [file Table_1.PDF]

Supplementary Table 1. Comparison of nimodipine AUC_{0-3h} based on patient outcomes.

	n	(-)-S nimodipine	(+)-R nimodipine	Total nimodipine
**mRS**				
mRS 90d 0-2 (all)	14	12 (7-15)	24 (17-100)	31 (28-125)
mRS 90d 3-6 (all)	9	14 (5-33)	32 (23-84)	38 (29-117)
p-value		0.85	0.45	0.66
mRS 90d 0-2 (high grade)	4	24 (8-35)	111 (44-158)	136 (52-192)
mRS 90d 3-6 (high grade)	6	6 (2-14)	23 (21-32)	33 (23-39)
p-value		0.14	0.20	0.20
mRS 90d 0-2 (low grade)	10	11 (7-13)	20 (17-27)	30 (28-36)
mRS 90d 3-6 (low grade)	3	53 (33-64)	119 (84-244)	172 (117-308)
p-value		**0.01**	**0.03**	**0.03**
**DCI**				
DCI (all)	7	13 (5-25)	64 (18-112)	78 (29-125)
No DCI (all)	17	11 (7-15)	25 (17-83)	36 (28-98)
p-value		0.82	0.63	0.68
DCI (high grade)	3	5 (2-14)	23 (6-64)	29 (8-78)
No DCI (high grade)	7	14 (2-33)	32 (21-138)	39 (23-175)
p-value		0.21	0.43	0.43
DCI (low grade)	4	19 (12-39)	106 (59-116)	125 (77-148)
No DCI (low grade)	10	11 (7-13)	24 (17-47)	31 (28-57)
p-value		0.12	0.12	0.12
**Vasospasm**				
Vasospasm (all)	12	12 (6-20)	25 (18-92)	37 (29-121)
No Vasospasm (all)	12	12 (7-24)	36 (19-111)	45 (28-136)
p-value		0.70	0.64	0.82
Vasospasm (high grade)	5	5 (2-6)	23 (6-24)	29 (8-38)
No Vasospasm (high grade)	5	15 (14-33)	83 (64-138)	98 (78-175)
p-value		0.08	**0.047**	**0.047**
Vasospasm (low grade)	7	13 (11-33)	84 (18-112)	117 (30-125)
No Vasospasm (low grade)	7	11 (7-13)	22 (16-47)	29 (27-57)
p-value		0.11	0.18	0.11

For modified Rankin Scale (mRS) at 90 days, n=23 (exclusions: 1 participant lost to follow-up; 1 participant did not have sufficient pharmacokinetic data and five were with delayed presentation > 96 h); for delayed cerebral ischemia (DCI) and vasospasm, n=24 (exclusions: 1 participant did not have sufficient pharmacokinetic data and five were with delayed presentation > 96 h).

Supplementary Table 2. Comparison of nimodipine C_{max} based on patient outcomes.

	n	Cmax S	Cmax R	Cmax total
**mRS**				
mRS 90d 0-2 (all)	14	4 (3-7)	10 (7-44)	15 (10-54)
mRS 90d 3-6 (all)	9	5 (3-16)	19 (11-38)	20 (16-54)
p-value		0.99	0.45	0.41
mRS 90d 0-2 (high grade)	4	12 (4-20)	59 (20-85)	71 (24-105)
mRS 90d 3-6 (high grade)	6	3 (1-5)	14 (9-19)	18 (12-20)
p-value		0.09	0.20	0.14
mRS 90d 0-2 (low grade)	10	4 (3-7)	8 (7-13)	13 (10-20)
mRS 90d 3-6 (low grade)	3	24 (16-40)	68 (38-118)	84 (54-158)
p-value		**0.01**	0.06	**0.03**
**DCI**				
DCI (all)	7	5 (3-10)	24 (7-68)	29 (10-84)
No DCI (all)	17	5 (3-8)	13 (8-34)	20 (12-41)
p-value		0.82	0.72	0.82
DCI (high grade)	3	3 (1-5)	9 (4-24)	12 (5-29)
No DCI (high grade)	7	5 (3-18)	19 (11-83)	20 (16-101)
p-value		0.21	0.21	0.21
DCI (low grade)	4	7 (4-17)	56 (25-104)	69 (32-113)
No DCI (low grade)	10	6 (3-8)	10 (8-33)	15 (10-41)
p-value		0.40	0.15	0.20
**Vasospasm**				
Vasospasm (all)	12	4 (3-8)	11 (6-41)	15 (10-54)
No Vasospasm (all)	12	6 (4-13)	22 (8-59)	25 (13-71)
p-value		0.33	0.45	0.45
Vasospasm (high grade)	5	3 (3-4)	9 (6-11)	12 (9-16)
No Vasospasm (high grade)	5	6 (5-18)	34 (24-83)	40 (29-101)
p-value		0.12	**0.009**	**0.02**
Vasospasm (low grade)	7	5 (4-16)	38 (7-68)	54 (10-84)
No Vasospasm (low grade)	7	7 (3-8)	9 (8-33)	14 (10-41)
p-value		0.75	0.41	0.41

For modified Rankin Scale (mRS) at 90 days, n=23 (exclusions: 1 participant lost to follow-up; 1 participant did not have sufficient pharmacokinetic data and five were with delayed presentation > 96 h); for delayed cerebral ischemia (DCI) and vasospasm, n=24 (exclusions: 1 participant did not have sufficient pharmacokinetic data and five were with delayed presentation > 96 h).
